# Biokinetics and Subchronic Toxic Effects of Oral Arsenite, Arsenate, Monomethylarsonic Acid, and Dimethylarsinic Acid in v-Ha-*ras* Transgenic (Tg.AC) Mice

**DOI:** 10.1289/txg.7152

**Published:** 2004-06-18

**Authors:** Yaxiong Xie, Kevin J. Trouba, Jie Liu, Michael P. Waalkes, Dori R. Germolec

**Affiliations:** ^1^Inorganic Carcinogenesis Section, Laboratory of Comparative Carcinogenesis, National Cancer Institute at the National Institute of Environmental Health Sciences and; ^2^Environmental Immunology, National Institute of Environmental Health Sciences, Research Triangle Park, North Carolina, USA

**Keywords:** arsenicals (arsenic forms), gene expression, mouse liver, subchronic toxicity, toxicokinetics

## Abstract

Previous research demonstrated that 12-*O*-tetradecanoylphorbol-13-acetate (TPA) treatment increased the number of skin papillomas in v-Ha-*ras* transgenic (Tg.AC) mice that had received sodium arsenite [(As(III)] in drinking water, indicating that this model is useful for studying the toxic effects of arsenic *in vivo*. Because the liver is a known target of arsenic, we examined the pathophysiologic and molecular effects of inorganic and organic arsenical exposure on Tg.AC mouse liver in this study. Tg.AC mice were provided drinking water containing As(III), sodium arsenate [As(V)], monomethylarsonic acid [(MMA(V)], and 1,000 ppm dimethylarsinic acid [DMA(V)] at dosages of 150, 200, 1,500, or 1,000 ppm as arsenic, respectively, for 17 weeks. Control mice received unaltered water. Four weeks after initiation of arsenic treatment, TPA at a dose of 1.25 μg/200 μL acetone was applied twice a week for 2 weeks to the shaved dorsal skin of all mice, including the controls not receiving arsenic. In some cases arsenic exposure reduced body weight gain and caused mortality (including moribundity). Arsenical exposure resulted in a dose-dependent accumulation of arsenic in the liver that was unexpectedly independent of chemical species and produced hepatic global DNA hypomethylation. cDNA microarray and reverse transcriptase–polymerase chain reaction analysis revealed that all arsenicals altered the expression of numerous genes associated with toxicity and cancer. However, organic arsenicals [MMA(V) and DMA(V)] induced a pattern of gene expression dissimilar to that of inorganic arsenicals. In summary, subchronic exposure of Tg.AC mice to inorganic or organic arsenicals resulted in toxic manifestations, hepatic arsenic accumulation, global DNA hypomethylation, and numerous gene expression changes. These effects may play a role in arsenic-induced hepatotoxicity and carcinogenesis and may be of particular toxicologic relevance.

Arsenic is an important environmental toxicant and carcinogen [International Agency for Research on Cancer (IARC) 1987; National Research Council (NRC) 1999]. Chronic exposure to arsenic via drinking water is a major health concern throughout the world (Gebel 2000; NRC 1999). The carcinogenic effects of environmental arsenic exposure in human populations are well documented (IARC 1987; NRC 1999), and exposure can lead to tumors in and toxicity of the skin, lung, urinary bladder, liver, and other sites.

The adverse effects of arsenic are dependent, in part, on its chemical form and metabolism (Aposhian 1997; Vahter 2002). Humans are exposed primarily to trivalent [arsenite, As(III)] and pentavalent [arsenate, As(V)] inorganic arsenicals present in the environment, as well as to organic arsenic [e.g., dimethylarsinic acid, DMA(V)] in some situations (Kenyon and Hughes 2001; Shen et al. 2003b). In mammals, As(V) is first reduced to As(III), whereas As(III), produced by this reduction or from direct ingestion, is methylated primarily to pentavalent organic arsenicals including monomethylarsonic acid [MMA(V)] and DMA(V)]. MMA and DMA are the predominant metabolites of inorganic arsenic (Vahter 2002), although DMA may be further methylated to trimethylarsine oxide (TMAO) (Hughes 2002; Yoshida et al. 1998). The forms of arsenic to which humans are exposed, either directly or via metabolism, further complicate the elucidation of their toxic and carcinogenic mechanisms of action. Previously, inorganic arsenicals were thought to be more acutely toxic than organic species, as the methylation of inorganic arsenic was proposed to be a detoxification process. However, recent studies indicate that trivalent organic arsenicals [e.g., MMA(III) and DMA(III)] that are metabolic products of inorganic arsenic can be more toxic than the parent compound (Petrick et al. 2001; Styblo et al. 2000). Furthermore, DMA can act as a tumor promoter at various sites and as a complete carcinogen for the urinary bladder in rats (Salim et al. 2003; Wei et al. 2002; Yamamoto et al. 1997). MMA produces preneoplastic changes in liver and urinary bladder but does not produce overt neoplasia (Shen et al. 2003a), whereas TMAO can induce hepatocellular adenomas (Shen et al. 2003b). Therefore, it is important to compare and evaluate the toxicity of As(III), As(V), MMA(V), and DMA(V) under similar experimental conditions.

Recent studies demonstrated that arsenic acts as a co-promoter with 12-*O*-tetradecanoylphorbol-13-acetate (TPA) because together they enhance skin tumor development in transgenic (Tg.AC) mice, which overexpress the v-Ha-*ras* oncogene (Germolec et al. 1997, 1998; Trouba et al. 2003). Because hepatic metabolism in Tg.AC mice is not compromised by over-expression of the v-Ha-*ras* oncogene (Sanders et al. 2001), we hypothesized that organic and inorganic arsenicals produce similar yet distinct changes in Tg.AC liver gene expression that may be predictive of hepatotoxicity. The latter is important because the liver is an important target organ of arsenic toxicity in animals (Waalkes et al. 2000b) and humans (Lu et al. 2001). The liver is also a major target organ of arsenic carcinogenicity after *in utero* exposure in mice (Waalkes et al. 2003, 2004b) and in humans exposed to environmental arsenic (Centeno et al. 2002; Zhou et al. 2002). To address the above hypothesis, we examined the effects of subchronic inorganic and organic arsenical exposure on the Tg.AC mouse liver. Our results indicate that in Tg.AC mice, *a*) hepatic arsenic [e.g., As(III), As(V), MMA(V), and DMA(V))] accumulation, based on biokinetic analyses, was dose dependent; *b*) global DNA hypomethylation occurred after exposure to As(III) As(V), MMA(V), and DMA(V); *c*) pathological changes were present in the liver after exposure to As(III), MMA(V), and DMA(V); and *d*) arsenic-induced gene expression changes, determined using cDNA microarray and real-time reverse transcriptase–polymerase chain reaction (RT–PCR) analysis, occurred in the liver of animals treated with As(III), As(V), MMA(V), and DMA(V).

## Materials and Methods

### Chemicals

As(III) and As(V) were purchased from Aldrich Chemical Co. (Milwaukee, WI) and Fluka Chemical Corp. (Milwaukee, WI), respectively. MMA(V) was obtained from AccuStandard, Inc. (New Haven, CT). DMA(V) and TPA were purchased from Sigma Chemical Co. (St. Louis, MO). Customer-designed cDNA micro-arrays (600 genes) were purchased from BD Biosciences Clontech, Inc. (Palo Alto, CA). [α-^32^P]-deoxyadenosine 5′-triphosphate was purchased from PerkinElmer, Inc. (Boston, MA), and ^3^H-labeled *S*-adenosylmethionine ([^3^H]-SAM) was from Amersham (Arlington Heights, IL).

### Animal Treatment

All animals were handled and treated in compliance with the *Guide for the Care and Use of Laboratory Animals* (NRC 1996). Female, homozygous Tg.AC mice containing the fetal zeta-globin promoter fused to the v-Ha-*ras* structural gene (with mutations at codons 12 and 59) and linked to a simian virus 40 polyadenylylation/ splice sequence were obtained from Taconic Farms (Germantown, NY) (Leder et al. 1990). Mice were maintained in an animal facility at a temperature of 20–22°C, a relative humidity of 50%, and a 12-hr light/dark cycle. Mice were randomly assigned to five groups (*n* = 15 in each group) and were provided unaltered drinking water (control) and drinking water containing As(III) (150 ppm as arsenic), As(V) (200 ppm as arsenic), MMA(V) (1,500 ppm as arsenic), and DMA(V) (1,000 ppm as arsenic), respectively, for 17 weeks. The doses of arsenicals used were based on our previous studies (Germolec et al. 1997, 1998). Multiple doses of each arsenical were originally used to examine papilloma development. However, to detect gene expression changes in the liver that may be related to arsenic hepatotoxicity and hepatocellular carcinogenesis, animals treated with the maximal dose of each arsenical were selected for analysis.

Four weeks after initiation of arsenic treatment, TPA at a dose of 1.25 μg/200 μL acetone was applied twice weekly for 2 weeks to the shaved dorsal skin of all mice, including the mice not receiving arsenic (control). At 17 weeks the mice were sacrificed by CO_2_ asphyxiation and necropsied. Liver tissue was excised and stored at −70°C until analysis or fixed for histology as described below.

During the exposure to arsenic, mortality, moribundity, clinical symptoms, body weight, and water intake of the mice were monitored. All mice, including those found deceased or sacrificed as moribund, underwent complete necropsy.

### Pathological Examination

Liver samples were fixed with neutral-buffered formalin, processed by standard procedures, embedded in paraffin, sectioned, and stained with hematoxylin and eosin for light microscopy examination. All pathological assessments were performed in a blind fashion.

### Hepatic Arsenic Levels

A portion of the frozen liver (120–150 mg) was digested in nitric acid. Total arsenic, which would include inorganic and organic forms, was determined using graphic furnace atomic absorption spectrometry (Perkin-Elmer AAnalyst100; PerkinElmer, Inc., Norwalk, CT). Results were expressed as micrograms arsenic per gram wet weight liver, as reported in our recent publications (Liu et al. 2001a; Xie et al. 2004).

### Global DNA Methylation Assay

Genomic DNA was extracted from liver tissue and purified using DNeasy Kits (Qiagen, Valencia, CA). Global DNA methylation status was assessed by methyl acceptance assay (Chen et al. 2004). Briefly, DNA (1 μg) was incubated at 37°C for 2 hr in a 30-μL mixture containing 1.25 μM (3 μCi) [^3^H]-SAM, 4 units CpG methylase (M. Sss I) (New England Biolabs, Inc., Beverly, MA), 10 mM DDT, Tris-EDTA buffer (100 mM Tris, 10 mM EDTA, pH 8.0), and 100 mM NaCl. The reaction was terminated on ice and transferred onto a Whatman DE81 filter (Whatman International Ltd., Maidstone, U.K.). The filter was washed on a vacuum filtration apparatus with 2 mL 0.5 M phosphate buffer (pH 7.0) 5 times, followed by a wash with 2 mL 70% ethanol and 2 mL absolute ethanol. After the filter was dried, the bound radioactivity was measured by scintillation (Beckman LS 6500 Scintillation Counter; Beckman Coulter, Inc., Fullerton, CA).

### cDNA Microarray Analysis

Microarray analysis was performed as previously described (Xie et al. 2004). Briefly, total RNA was extracted from liver tissues with Trizol reagent and purified with RNeasy columns (Qiagen). Five micrograms pooled RNA (*n* = 5) was converted to [α-^32^P]-dATP–labeled cDNA probe with Atlas specific cDNA synthesis primers (BD Biosciences Clontech Inc.). The probe was purified with a NucleoSpin column (BD Biosciences Clontech), denatured at 100°C for 2–3 min, and hybridized to the membrane in triplicate with Expresshyb buffer (BD Biosciences Clontech) at 68°C overnight. The membranes were washed at 68°C four times (30 min each) in 2 × sodium chloride/sodium citrate (SSC)/1% sodium dodecyl sulfate (SDS), twice in 0.1 × SSC/0.5% SDS, and exposed to a phosphoimage screen. Images were acquired by PhosphorImager Scanner (Model Storm 860; Molecular Dynamics, Sunnyvale, CA) and analyzed densito-metrically using AtlasImage software (version 2.01; Clontech).

### Real-time RT–PCR Analysis

Total RNA was reverse transcribed with MMLV reverse transcriptase and oligodT primers (PerkinElmer Inc.). The PCR primers were designed with Primer Express software and the SYBR Green DNA PCR kit (Applied Biosystems, Foster City, CA) was used for real-time RT–PCR analysis. Differences in gene expression between groups were calculated using cycle time (Ct) values, which were normalized against β-actin and expressed as relative increases/ decreases, setting control as 1.0. Assuming that the Ct value is reflective of the initial template amount (copy number) and that there is 100% efficiency, a difference of one cycle is equivalent to a 2-fold difference in initial copy number (Walker 2001).

### Statistics

Data are expressed as mean ± SEM or as incidence (for mortality). For comparisons of gene expression between two groups, the Student *t* test was used. For comparisons among three or more groups, data were analyzed using a one-way analysis of variance, followed by Duncan’s multiple range test. The *p*-value was calculated by Fisher’s exact test for incidence data. The level of significance was set at *p* < 0.05 in all cases. Two-dimensional hierarchical cluster analysis of microarray data (hybrid intensity ratios to control values) was performed. The results from clustered analysis were examined by interactive graphical analysis using TreeView software (http://rana.lbl.gov/EisenSoftware.htm).

## Results

### Clinical Symptoms

**Figure 1:**
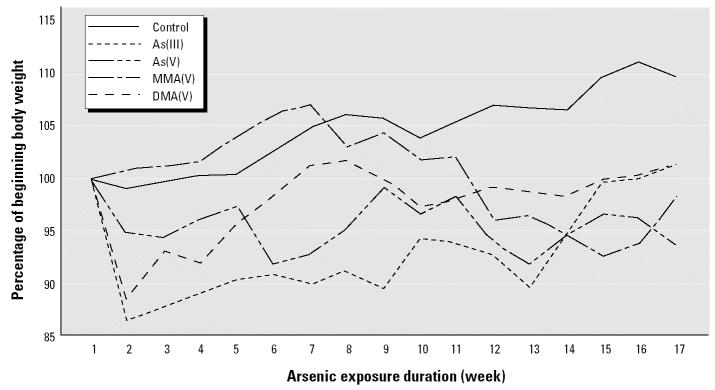
Change in animal body weight during exposure to arsenicals.

During the 17 weeks of arsenical exposure, several arsenic-treated mice were found deceased or were euthanized because of moribundity. Exposure to As(III) (150 ppm) and DMA(V) (1,000 ppm) resulted in 20% mortality, and exposure to MMA(V) (1,500 ppm) resulted in 40% mortality (Table 1). MMA(V) exposure alone produced significant toxicity when compared with control. In general, the body weight in arsenic-treated groups was lower than that in control groups. At the end of arsenic exposure (17 weeks), body weight was decreased by approximately 15, 8, 10, and 8% in mice treated with As(III) (150 ppm), As(V) (200 ppm), MMA(V) (1,500 ppm), or DMA(V) (1,000 ppm), respectively (Figure 1). Our findings suggest that exposure of Tg.AC mice to these arsenicals produced mild to moderate [for the MMA(V) group] toxicity.

### Pathology

**Figure 2:**
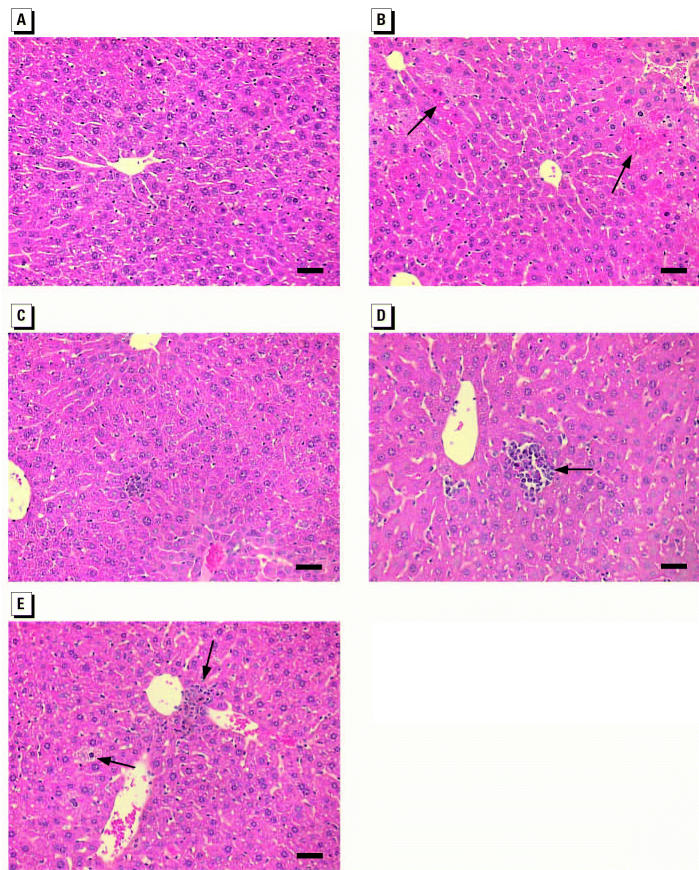
Hepatic pathological changes induced by arsenicals. (*A*) Control; (*B*) As(III), 150 ppm; (*C*) As(V), 200 ppm; (*D*) MMA(V), 1,500 ppm; (*E*) DMA(V), 1,000 ppm. Original magnification, ×200; bars = 50 μm.

The treatment of As(III) plus TPA did not induce liver tumor formation in Tg.AC mice treated with arsenicals for 17 weeks (unpublished data). However, morphologic changes including inflammation, foci of apoptosis and necrosis, and hepatocellular degeneration were observed in arsenic-treated mice (Figure 2). Foci of apoptosis and necrosis were observed in animals treated with As(III) (150 ppm); however, no apparent histologic alterations were present in animals that received As(V) (200 ppm). MMA(V) (1,500 ppm) produced inflammatory cell infiltration, degeneration, and swelling; DMA(V) (1,000 ppm) produced foci of inflammation and hepatocellular degeneration. These findings indicate that subchronic arsenical exposure produces pathological alterations in the liver.

### Hepatic Arsenic Content

**Figure 3:**
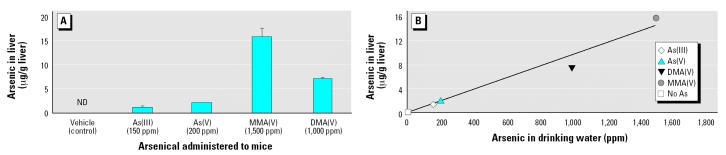
Hepatic arsenic content expressed as micrograms per gram wet weight. ND, not detected. (*A*) Arsenical species; (*B*) dose relationship.

Although not detectable in livers of controls, arsenic was found in the livers of all treatment groups (Figure 3). Particularly high levels of arsenic were present in the livers of the MMA-treated group (Figure 3A). When hepatic arsenic content was plotted against arsenical dose, a strong linear correlation was observed (*r* = 0.98) (Figure 3B), suggesting that subchronic arsenic exposure results in arsenic accumulation in the liver that is dose dependent.

### Global DNA Methylation Status

**Figure 4:**
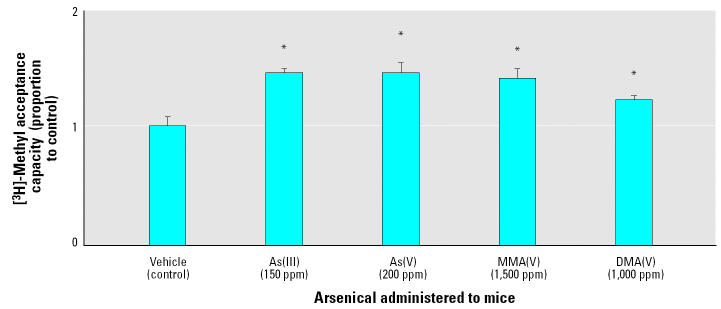
Methyl acceptance capacity of hepatic genomic DNA of mice exposed to arsenicals.
*Statistically significant (*p* < 0.05) compared with vehicle alone treatment (control).

Global DNA methylation was assessed by methyl acceptance assay (Figure 4). This assay uses a bacterial DNA methyltransferase that indiscriminately methylates all unmethylated cytosines using [^3^H]-SAM. Thus, higher [^3^H]-SAM incorporation corresponds to a lower degree of methylation (i.e., hypomethylation) of cellular DNA. The amount of unmethylated DNA from all the arsenical-treated groups was significantly higher (*p* < 0.05) than control, indicating that DNA hypomethylation occurs in the Tg.AC mouse liver after subchronic exposure to arsenic, regardless of the chemical form. When this is correlated with actual arsenic dose, As(III) is the most potent hypomethylating agent; MMA(V) is the least.

### Genomic Analysis by cDNA Microarray

**Figure 5:**
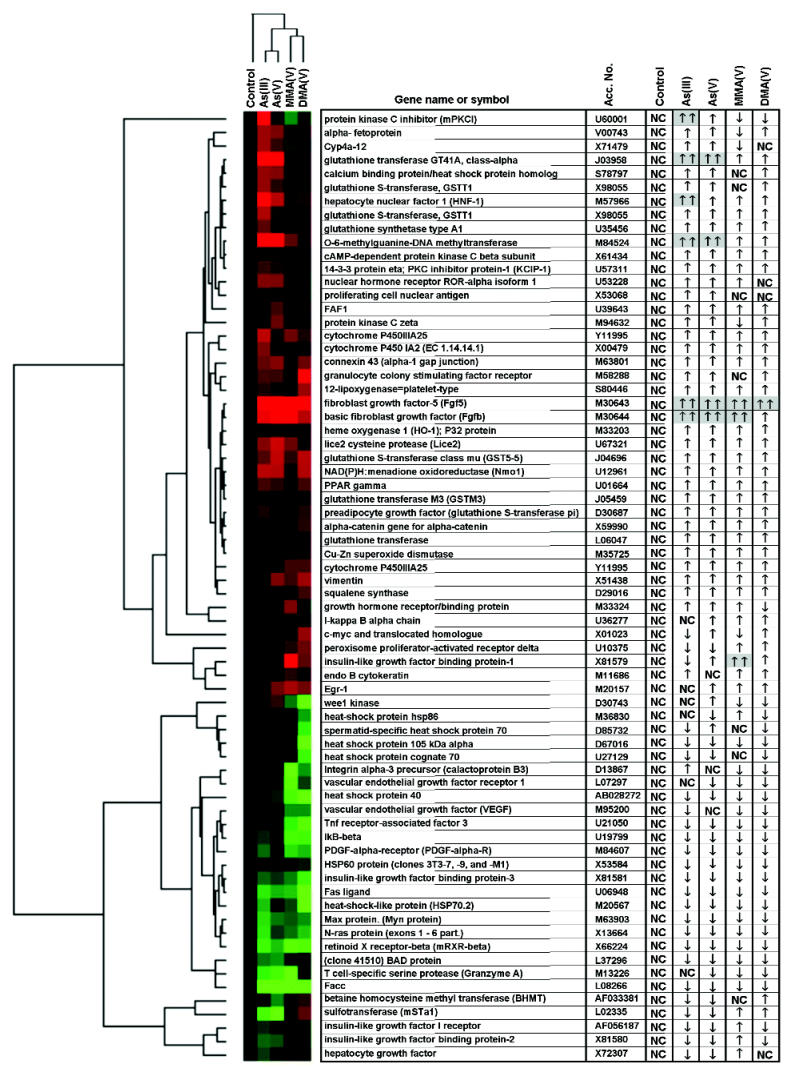
Cluster analysis of cDNA microarray data of selected genes. Data are ratio of control values: [red] ratio > 1.0; [black] ratio = 1.0; [green] ratio < 1.0. Relative changes in gene expression compared with those of control are presented as increased (↑), decreased (↓), or no change (NC). Double arrows highlighted in gray indicate ratio ≥2. Gene names and accession numbers are from GenBank (http://www.ncbi.nlm.nih.gov/entrez/query.fci?db=nucleotide).

Among the 600 genes examined via microarray analysis, 70 displayed increased or decreased expression after subchronic arsenic exposure. The hybrid intensity (ratio to control value) for these 70 genes was calculated for comparison then subjected to cluster analysis to compare alterations in gene expression patterns related to the type of arsenical exposure. TreeView revealed both similar and dissimilar changes in gene expression patterns among the four arsenicals (Figure 5).

The most significant arsenic-induced changes in gene expression are listed in Table 2. Genes associated with glutathione *S*-transferase (GST) function/metabolism, stress, apoptosis, cell proliferation, and early neoplasia are thought to be related to arsenic toxicity (Liu et al. 2004; Trouba et al. 2002, 2003; Xie et al. 2004) and thus are included for comparison. For example, all arsenicals produced increases in GSTs (alpha, mu, pi, and theta) and fibroblast growth factor 2, a gene related to cell proliferation. A significant increase in the expression of insulin-like growth factor binding protein 1 (IGFBP-1) also was found in MMA-treated mice. In general, all of the arsenicals produced similar effects (i.e., increase/decrease) on gene expression; however, the degree of change was different in some cases.

### Real-Time RT–PCR Analysis

**Figure 6:**
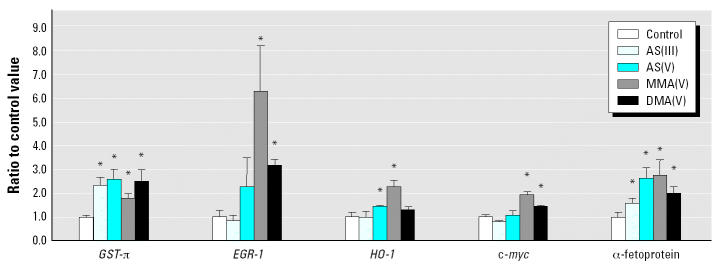
Real-time RT–PCR analysis of selected genes. Data are mean ± SEM (*n* = 5). Dosage (ppm as arsenic) of the arsenicals: As(III), 150 ppm; As(V), 200 ppm; MMA(V), 1,500 ppm; DMA(V), 1,000 ppm.
* Statistically significant (*p* < 0.05) compared with control.

Real-time RT–PCR analysis was performed for selected genes in each cluster. Figure 6 shows data for some of the genes of interest. *GST-*π, early growth response protein 1 (*EGR-1*), heme oxygenase 1 (*HO-1*), c-*myc*, and α-fetoprotein gene expression was enhanced after arsenical exposure. Generally, real-time RT–PCR analysis confirmed our microarray results.

## Discussion

This study demonstrated that subchronic exposure of transgenic (Tg.AC) mice to both inorganic and organic arsenicals through drinking water produced various effects on the liver, a major target organ of arsenic toxicity and carcinogenesis (Centeno et al. 2002; NRC 1999; Waalkes et al. 2003, 2004b). Arsenic-induced toxicity was evidenced by an increase in moribundity and death, a depression in body weight, hepatic pathological changes, and significant changes in gene expression.

An original goal of our research was to examine the effects of inorganic and organic arsenic on TPA-promoted skin papilloma development in Tg.AC mice. Although TPA was administered to all mice (including controls that received no arsenic), the effects of this skin tumor promoter were not deemed critical to our analyses of liver pathology, DNA methylation, and gene expression. Interestingly, topical application of TPA in some experimental models has systemic effects; we recently found that it promoted liver tumors initiated by transplacental arsenic exposure in female mice (Waalkes et al. 2004b). In this study epidermal TPA treatment resulted in no mortality and did not affect hepatic pathology, indicating that the biological end points/ changes measured are most likely dependent on arsenical treatment alone.

Because the liver is a major target organ of arsenic toxicity and carcinogenesis (Waalkes et al. 2000b, 2003, 2004b), we examined gene expression as well as pathological changes in the livers of Tg.AC mice to further explore the usefulness of this system as an *in vivo* model of arsenic carcinogenesis and toxicity. To detect gene expression changes that may be related to arsenic toxicity, animals treated with the maximal dose of each arsenical were selected for analysis. Generally, 150 ppm As(III) produced more toxicity and more dramatic changes in gene expression than 200 ppm As(V). Organic arsenicals at doses [1,500 and 1,000 ppm as arsenic for MMA(V) and DMA(V), respectively] 5-to 10-fold higher produced toxic effects comparable to those produced by As(III). Although rats are tolerant to 200 ppm MMA(V) in drinking water for 104 weeks (Shen et al. 2003a), the mice in our study did not tolerate MMA(V) at 1,500 ppm, as 40% mortality (i.e., moribundity and death) occurred in these mice over the 17-week exposure period. The dose of DMA(V) in this study was also higher than the doses (50 and 200 ppm) used to induce urinary bladder tumors in rats (Wei et al. 2002) and also exceeded the maximum tolerated dose, as it produced 20% mortality.

In our study, promoted and nonpromoted, arsenic-treated Tg.AC mice did not display direct evidence of liver tumor formation. However, preneoplastic lesions (e.g., cell proliferation) occur in the liver after chronic oral arsenic exposures in several strains of mice (Chen et al. 2004; Shen et al. 2003a; Waalkes et al. 2000b) and were also observed in the liver of Tg.AC mice exposed to arsenic in this study. Exposure to arsenic in the drinking water resulted in a dose-dependent accumulation of arsenic in the liver that was independent of chemical form. The highest hepatic content, which was observed in the high-dose (1,500 ppm) MMA(V) group, might contribute to the high degree of mortality (40%) in this group. The hepatic arsenic contents in the Tg.AC mice receiving 150 ppm As(III) and 200 ppm As(V) in this study were 1.2 and 2.0 μg/g tissue, respectively. This was less than the arsenic content in the skin (8.3 μg/g tissue) and much less than that in the hair (170.2 μg/g tissue) of Tg.AC mice exposed to 200 ppm As(III) in the drinking water for 14 weeks in our previous study (Germolec et al. 1998), indicating that arsenic accumulation in the liver is lower than that in the hair or skin. This may be because liver is the major target organ for arsenic metabolism, and arsenic elimination generally occurs through the bile (Gregus et al. 2000) or urine.

DNA hypomethylation occurs after chronic arsenic exposure in cells (Zhao et al. 1997) and also in intact animals (Chen et al. 2004; Okoji et al. 2002). In the present study, all arsenicals produced significant DNA hypomethylation in the liver, regardless of dose. Although the doses of MMA(V) (1,500 ppm) and DMA(V) (1,000 ppm) used in our study were much higher than those of As(III) (150 ppm) and As(V) (200 ppm), MMA(V) and DMA(V) induced less hypomethylation of hepatic DNA than As(III) and As(V). This suggests that inorganic arsenicals are more potent stimulators of DNA hypomethylation compared with MMA(V) and DMA(V). It should be noted that global DNA hypomethylation could co-exist with regional or individual gene hypermethylation, as arsenic-induced p53 hypermethylation has been reported (Mass and Wang 1997). In our recent study, we proposed that arsenic-induced hypomethylation of the estrogen receptor-α gene plays an important role in hepatocellular proliferation (Chen et al. 2004; Waalkes et al. 2004a). Efforts are currently being undertaken to examine the methylation status of individual genes after arsenic exposure.

DNA hypomethylation is an important mechanism involved in aberrant gene expression and carcinogenesis (Baylin et al. 1998; Goodman and Watson 2002). In particular, it is thought that aberrant DNA methylation is central to the development of liver cancers (Goodman and Watson 2002) and is an epigenetic mechanism that underlines the aberrant expression of genes involved in mouse liver carcinogenesis (Counts et al. 1997). In the present study, As(III), As(V), MMA(V), and DMA(V) produced variable gene expression changes, accounting for approximately 10% of genes on the array. We focused primarily on a few categories, for example, glutathione (GSH)-, apoptosis-, and cell proliferation–related genes, and genes important for tumor development, as previous studies have shown these to be related to aberrant cell growth and neoplasia.

Glutathione systems play important roles in arsenic toxicity and carcinogenesis (NRC 1999; Trouba et al. 2002, 2003; Xie et al. 2004). In the present study, the expression of GST-μ, GST-π, GST-α, and GST-τ was increased by all arsenicals, although to a variable extent. GSTs are a group of enzymes catalyzing the conjugation and oxidation of GSH with arsenic (Xie et al. 2004). An increase in GST expression/activity (particularly GST-π) has been reported to play an important role in cellular efflux of arsenic–GSH conjugates and to be a mechanism of arsenic tolerance (Brambila et al. 2002; Liu et al. 2001a; Wang et al. 1996). Increases in GST-π positive foci have been proposed to be a hepatic preneoplastic biomarker in chronic arsenic-exposed populations (Nishikawa et al. 2002; Shen et al. 2003a). Changes in GST activity in humans also are associated with altered arsenic metabolism (Chiou et al. 1997; Marnell et al. 2003), and GST polymorphisms are thought to be a susceptibility factor for arsenic toxicity in humans (Marnell et al. 2003). Together, these data indicate that increases in GST gene expression and/or function are consistent events associated with arsenic carcinogenicity and toxicity.

Oxidative stress is proposed to play an important role in arsenic toxicity and carcinogenesis (Kitchin and Ahmad 2003; Liu et al. 2001b; NRC 1999; Trouba et al. 2002; Xie et al. 2004). In addition to GSTs, other biomarkers for arsenic-induced oxidative stress such as HO-1 (Del Razo et al. 2001; Liu et al. 2001b), EGR-1 (Liu et al. 2004; Simeonova et al. 2000), DT- diaphorase (Pi et al. 2003), and cytochrome P450 3A25 (Liu et al. 2001b) were all increased in Tg.AC mice after exposure to arsenicals. Evidence is accumulating regarding the ability of arsenicals to produce reactive oxygen species and free radicals as measured using electron spin response. This includes inorganic As(III) and As(V) (Barchowsky et al. 1999) and organic MMA(III), DMA(III) (Nesnow et al. 2002), and DMA(V) (Yamanaka et al. 1990). Our data (e.g., gene expression changes and pathology) lend further evidence for the presence of oxidative stress during subchronic exposure to arsenicals.

Arsenic induces apoptosis involved in its mechanism of acute toxicity (NRC 1999). However, after chronic arsenic exposure and the induction of malignant transformation, the development of apoptosis resistance occurs (Brambila et al. 2002; Qu et al. 2002) and is associated with the downregulation of apoptosis-related genes (Brambila et al. 2002; Chen et al. 2001a). In the present study, arsenical exposure resulted in downregulation of apoptosis-associated genes such as *FasL*, tumor necrosis factor receptor–associated factor 3, *Bad*, and granzyme A, and also increased the expression of cell proliferation–related genes including c-*myc*, proliferating cell nuclear antigen, and fibroblast growth factor 2. These data are interesting in light of evidence that apoptosis tolerance and cell proliferation are important mechanisms involved in chemical carcinogenesis (Waalkes et al. 2000a), including arsenic. Apoptosis tolerance also is accompanied by cell proliferation, as seen in arsenic-transformed cells (Brambila et al. 2002; Chen et al. 2001b; Qu et al. 2002) in chronic arsenic-exposed animals (Chen et al. 2004; Xie et al. 2004), and in liver tumor and nontumor tissues from mice exposed to arsenic *in utero* (Liu et al. 2004; Waalkes et al. 2003). Thus, the depression of apoptosis genes and the overexpression of cell proliferation genes could be important in arsenic toxicity and carcinogenesis.

Liver is a major target of arsenic carcinogenesis in transplacentally exposed animal models (Waalkes et al., 2003) and in arsenic-exposed humans (Centeno et al. 2002; Chen et al. 1997; Zhou et al. 2002). The expression of α-fetoprotein (*AFP*), a biomarker for hepatocellular carcinogenesis, was increased in transplacental arsenic-induced hepatocellular carcinoma (HCC) and tumor-surrounding tissues (Liu et al. 2004). In the present study, all the arsenicals tested increased *AFP* expression up to 3-fold in MMA(V)-treated mice. The enhanced expression of *AFP* lends further support that preneoplastic alterations occur after subchronic arsenic exposure. Other notable alterations in gene expression were the overexpression of *IGFBP-1* and suppression of insulin-like growth factor 1 (*IGF-1*). Chronic exposure to nongenotoxic chemicals such as oxazepam and Wyeth-14,643 increased the expression of *IGFBP-1* in a time-dependent manner (Iida et al. 2003), and overexpression of *IGFBP-1* was also seen in transplacental arsenic-induced HCC and tumor-surrounding tissues (Liu et al. 2004). Dysregulation of the IGF axis has been implicated in liver tumor formation and progression (Scharf et al. 2001). Thus, sub-chronic exposure to arsenicals can produce aberrant gene expression related to hepatocarcinogenesis, some of which were confirmed in the present study.

In summary, this study demonstrated that subchronic exposure to As(III), As(V), MMA(V), and DMA(V) in the drinking water resulted in variable toxic effects, accumulation of arsenic in the liver, hepatic global DNA hypomethylation, and alterations in gene expression in Tg.AC mice. These findings indicate that liver is a target organ of subchronic arsenical exposure in this model and support the idea that altered DNA methylation and its effects on gene expression may contribute in an epigenetic manner to arsenic carcinogenesis.

## Figures and Tables

**Table 1:** Mortality due to arsenical exposure.*^a^*

Arsenical	No.	Found dead	Sacrificed moribund	Total (%)*^b^*
Control	15	0	0	0 (0)
AS(III) (150 ppm)	15	1	2	3 (20)
As(V) (200 ppm)	15	0	0	0 (0)
MMA(V) (1,500 ppm)	15	3	3	6 (40)*
DMA(V) (1,000 ppm)	15	2	1	3 (20)

a Arsenicals (ppm as arsenic) were administered for 17 weeks in drinking water; animal health was monitored twice daily.

b Total dead or euthanized before end of the experiment.

* Statistically significant (*p* < 0.05) compared with control.

**Table 2:** Effect of arsenicals on selected gene expression.*^a^*

			Intensity relative to that of control			
Protein/gene	Accession no.*^b^*	Hybrid intensity of control	As (III) (150 ppm)	As (V) (200 ppm)	MMA (V) (1,500 ppm)	DMA (V) (1,000 ppm)
GST gene						
*GST-alpha*	J03958	5,306	2.38*	2.25*	1.23*	1.17
*GST-mu*	U24428	3,824	1.52	1.83*	1.43	1.82*
*GST-pi*	D30687	25,368	1.40*	1.25	0.96	1.40
*GST-theta-1*	X98055	3,892	1.70*	1.42	1.03	1.12
Stress-related genes						
*HO-1*	M33203	13,480	1.19	1.31*	1.31*	1.26*
*EGR-1*	M20157	6,356	0.88	1.59*	1.76*	1.61*
DT diaphorase	U12961	2,926	1.81*	1.79*	1.28	1.79*
Cytochrome P450 IIIA25 (CYP3A25)	Y11995	19,041	1.22	1.21	1.48*	1.47*
Genes related to apoptosis and cell proliferation						
*FasL*	U06948	2,006	0.60	0.65	0.63	0.33*
TNF receptor–associated factor 3	U21050	3,452	0.81	0.82	0.58*	0.49*
*Bad*	L37296	3,002	0.70*	0.67*	0.81	0.92
Granzyme A	M13226	2,802	0.58*	0.53*	0.42*	0.90
Proliferating cell nuclear antigen	X53068	2,768	1.33*	1.21	0.99	0.96
Fibroblast growth factor 2	M30644	1,582	2.00*	2.19*	2.16*	1.80*
*c-myc* protooncogene	X00195	1,981	1.18	1.11	0.93	1.71
Tumor-related genes						
Alpha fetoprotein	V00743	1,631	1.72*	1.63	0.88	1.28
Insulin-like growth factor binding protein 1	X81579	7,605	0.94	1.20	2.93*	1.54*
Insulin-like growth factor 1	AF056187	1,216	0.65	0.95	0.23	0.47

a Data are based on the average value of arrays run in triplicate.

b Accession numbers are from GenBank (http://www.ncbi.nlm.nih.gov/entrez/query.fci?db=nucleotide).

* Original hybrid intensity is significantly different from that of control (*p* < 0.05).
